# Community Engagement Leads to Professional Identity Formation of Nursing Students

**DOI:** 10.31372/20200503.1105

**Published:** 2020

**Authors:** Edna R. Magpantay-Monroe, Ofa-Helotu Koka, Kamaile Aipa

**Affiliations:** Chaminade University of Honolulu, Hawaii, United States

**Keywords:** professional identity, nursing students, community engagement, mentoring

## Introduction

Professional identity formation is essential to nursing education. Knowledge, skills, attitudes, and values help form nursing students’ identity. Professional identity is a process of becoming independent and having self-awareness of one’s educational journey (All Answers Ltd., 2018). Maranon and Pera (2015) described that the contrast between didactic and clinical learning may play a role in the ambiguity that initiates nursing students about professional identity. There is a gap in the current research literature and has been underexplored with no intentional plan to address new areas (Godfrey, 2020; Haghighat, Borhani, & Ranjbar, 2020). The goal of professional identity formation is to develop well-rounded students with moral competencies who will blossom into future nursing leaders (Haghighat et al., 2020). The benefit to the community of producing well-rounded nursing students is safety and quality in their actions. This descriptive paper will address examples of how professional identity may be achieved by nursing students’ participation in community engagement such as attendance to professional conferences and intentional mentoring.

## Significance

Students are encouraged to attend professional conferences to expand their knowledge and network with other nursing professionals. This attendance is vital to their beginning formation of their professional identity. Being an active participant of professional conferences allow students to be a part of conversations with those who have practiced in different aspects of nursing, which can be translated as a feeling of belonging (Silva, de Freitas, Takashi, & de Araújo Albuquerque, 2019). Students are exposed to situations that help them think more critically and professionally; guiding their actions as future nurses. These actions include, but are not limited to, considering new career goals, becoming more familiar with different types of nursing skills, or learning about new research topics that are being studied within the healthcare field. Most students’ attendance to professional conferences are funded through their school’s scholarship monies.

Participation in nursing professional development is rewarding and provides nursing students a view of the application to the nursing profession beyond the classroom (Maranon & Pera, 2015). This participation often does not happen without faculty mentoring. Faculty mentoring allows for processing of knowledge, skills, attitudes, and values that require clarification for students. The faculty mentoring helps minimize the dissonance of nursing students’ values to the real-world experience (Hite & Godfrey, 2019).

## Description of Community Engagement

Two nursing students who are Ho‘oulu Scholars attended the Quality and Safety Education for Nurses (QSEN) conference in May 2019 with their faculty mentor. One student is Native Hawaiian and Pacific Islander and the other student is Native Hawaiian and Portuguese; both of whom volunteered to attend the conference as part of their professional development funding through their scholarship. Both students are 21-year-old females from Maui and attend a private Catholic university on O?ahu.

QSEN is a national initiative to integrate knowledge, skills, and attitudes (KSAs) in nursing education for the purpose of safety and quality of healthcare (Disch, 2012; Dolansky & Moore, 2013). This conference was chosen by the faculty mentor as the faculty mentor is part of a national multisite study, who had a presentation scheduled at the conference. In addition, the timing of the conference which took place after the school semester ended allowed for students and faculty to travel without so many obligations.

The Ho‘oulu Scholarship is funded through a collaboration between Chaminade University of Honolulu and Kamehameha Schools whose “vision is to train a new generation of Hawaiian scientists, health practitioners, forensic specialists, environmental professionals, and business leaders who are grounded in science and culture. These scholars are promising young scientists who are groomed to lead the change for a healthy, prosperous, just, and sustainable l?hui (community) of the future” (Chaminade University of Honolulu, 2020, para 1). Students are chosen based on their commitment to the future of Hawai‘i and who may need transformative levels of financial, academic, and personal support (Chaminade University of Honolulu, para 2). The student scholars are “1. passionate about science, technology, and seeking solutions to humanity’s biggest challenges, 2. driven to explore a science education enriched by Hawaiian and Pacific culture and knowledge and 3. attracted to cutting edge majors such as nursing, pre-med, data science, community and public health…” (Chaminade University of Honolulu, para 3).

The two students were encouraged by their faculty mentor to share their learning from the conference with the community. The students shared their learning with the community in two ways. The video and poster presentation were chosen because these provided a good avenue of dissemination and familiarity to the students. First, students created a video about the connection between evidence-based practice in informatics and safety. Students explained the value of compassionate evaluation by clinical adjuncts to encourage students to improve their clinical performance to be safe and provide quality patient centered care. The video was presented to nursing adjuncts who taught at the school where students were matriculated. Second, students presented a poster at a local nursing conference outlining their learning moments at the QSEN conference. The topics of interest at the local conference included any information related to education and clinical nursing. The conference is considered an Education Day for one local nursing organization. Student presenters were encouraged. Students list the benefits of applying QSEN to their day-to-day role as future nurses as follows: (1) Allows students the opportunity to learn in a safe and blame-free environment; (2) Allows for reflection and prevention of future mistakes; (3) Develop further critical thinking skills; (4) Learn effective team collaboration; and (5) Standardized care for maximum equality and development of compassionate care. These learning moments help nursing students to incorporate safe and compassionate care into their professional identity.

## Outcomes of Community Engagement

A strong foundation for nursing students is formed through community engagement, which allows successful transition and positive outcomes toward the formation of professional identity. It is through their community engagement of presenting at a conference, sharing their learning to the school community, and engagement in mentoring of their faculty that hopefully helps these nursing students to develop their professional identity.

The video presentation included a Question and Answer (Q & A) portion. Participants such as clinical adjuncts and full-time faculty showed appreciation of the new knowledge through anecdotal comments and even offered examples from their experiences. The poster presentation provided some growth in the student’s experiential learning and allowed dialogue with community of interest like the clinical adjuncts, clinicians, educators, and other nursing students. At the local conference, the participants and education committee commented verbally to the faculty mentor and students regarding their satisfaction of the presentation through their engagement during the poster presentation session as students were able to answer questions and offer insights of their learning.

Stepping outside of one’s personal bubble allows for a greater view of nursing practices and their impact on patients. When students take advantage of educational and enrichment opportunities, they allow themselves to open their minds and adapt to new perspectives, which mold them into more well-rounded and mindful individuals. This process is not easy for any nursing student without intentional informal or formal mentoring. Nursing faculty should always aspire to role model professional behaviors in a variety of ways and provide nursing students with multiple opportunities to hone their professional identity. Professional mentoring of students is important because mentoring models professional behaviors. Mentoring gives students the confidence to endeavor to be a part of a larger community. Faculty mentors by clarifying knowledge that may not be familiar with the students, role modeling the process of networking, engagement in opportunities to present to a community that can yield new knowledge and emulating professional behaviors that build professional relationships.

## Discussions and Recommendations

Professional identity formation through reflective practice and intentional inquiry is important for nursing students (Haghighat, Borhani, & Ranjbar, 2020). Students, in a short period of their nursing education, were able to attend a nationally recognized conference, present their learning to the community through two avenues (i.e., video and poster presentation) and were mentored by a faculty member throughout this process. These accomplishments not only show positive development of knowledge, skills, and the formulation of best practices, but also inspires a changing culture of attitude which may suggest formation of professional identity. Recognizing past shortcomings in a culture of blame, students blossom to embrace a just culture of open communication, acceptance, and compassion. Learning to be accepting helps students to learn more effectively, understand deeply, prevent future mistakes, and be honest with themselves and their team. Attending a QSEN conference was significant to the students’ professional identity formation.

Students verbalized the following positive outcomes in relation to their community engagement: (1) the engagement brought awareness of new and credible information concerning patient safety to the community of healthcare providers and (2) the engagement brought feelings of empowerment and an open mind that reaffirms the abundance of possibilities. A ripple effect or a chain reaction was created. Empowering creates student leaders capable of change who take knowledge back to their communities.

Mentoring the two students allowed deeper engagement that happens beyond the classroom to help students gain confidence in their abilities as future nurses. The mindful development of professional nursing values, values that are morally and ethically in accordance with safe nursing practice is hopeful (Haghighat et al., 2020). Being a moral, ethical, and safe nurse is a strong contribution to their community as healthcare providers. Faculty mentors must emulate proper etiquette of networking with other healthcare professionals, demonstrate advocacy in the cause for safe and quality nursing, and allow students’ voices to be heard to elevate their cause.

One of the limitations of this initiative is the number of students that can be funded to go to a national professional nursing conference. The monetary award for nursing student professional development is limited. Sources of funding sometimes limits who is eligible. Another limitation is the time frame of the conference as some conference fall during the busiest time of the semester and students are hesitant to volunteer. Another limitation is the availability of faculty time to mentor students due to faculty’s own obligations to their professional development.

The recommendations for nursing schools are to encourage participation of nursing students in professional conferences through help with funding, to allow students to learn and give back to their community of peers through presentations, to encourage student champions in specific areas of interests such as safety, and to role model effective networking and professional presentations by encouraging their faculty to engage in mentorship. Formal or informal mentoring of nursing students by faculty to create a professional identity that is congruent to the mission of the school aids students in their vision of becoming competent, compassionate, and community building nurses. Future studies or projects can include survey of current student body at different life cycle of their nursing program to assess the knowledge, skills, and attitudes development that may have helped their professional identity. A more robust way of looking at identity formation signals adaptability with the changing healthcare environment.

## Acknowledgments

The author thank Sarah Powelson, MS, RN for her editing expertise and Chaminade University of Honolulu Ho‘oulu scholarship for funding the students’ attendance to the QSEN conference.
